# The Recovery of Weak Impulsive Signals Based on Stochastic Resonance and Moving Least Squares Fitting

**DOI:** 10.3390/s140813692

**Published:** 2014-07-29

**Authors:** Kuosheng. Jiang, Guanghua. Xu, Lin. Liang, Tangfei. Tao, Fengshou. Gu

**Affiliations:** 1 School of Mechanical Engineering, Xi'an Jiaotong University, Xi'an 710049, China; E-Mails: jiangkuosheng333@stu.xjtu.edu.cn (K.J.); taotangfei@mail.xjtu.edu.cn (T.T.); 2 State Key Laboratory for Manufacturing System Engineering, School of Mechanical Engineering, Xi'an Jiaotong University, Xi'an 710049, China; 3 Key Laboratory of Education Ministry for Modern Design and Rotor-Bearing System, Xi'an 710049, China; 4 School of Computing and Engineering, University of Huddersfield, Queensgate, Huddersfield HD1 3DH, UK; E-Mail: F.Gu@hud.ac.uk

**Keywords:** weak impulsive signals, parameter-tuning stochastic resonance, moving least squares fitting, recovery

## Abstract

In this paper a stochastic resonance (SR)-based method for recovering weak impulsive signals is developed for quantitative diagnosis of faults in rotating machinery. It was shown in theory that weak impulsive signals follow the mechanism of SR, but the SR produces a nonlinear distortion of the shape of the impulsive signal. To eliminate the distortion a moving least squares fitting method is introduced to reconstruct the signal from the output of the SR process. This proposed method is verified by comparing its detection results with that of a morphological filter based on both simulated and experimental signals. The experimental results show that the background noise is suppressed effectively and the key features of impulsive signals are reconstructed with a good degree of accuracy, which leads to an accurate diagnosis of faults in roller bearings in a run-to failure test.

## 1. Introduction

An impulsive signal is a typical vibration response due to faults in many mechanical components such as bearings and gears. It is characterized by the presence of a periodic repetition of sharp peaks modulated by high frequency harmonic components, which are defined in terms of natural frequency, fault frequency and decay coefficient [1]. In mechanical systems, a regularity of impulsive signals may arise from rotor-to-stator rub, defects or wear of certain parts such as bearings and gears. Impulsive signals can also be generated by many other mechanisms and can be found in many other applications. Acoustic noise and image noise are typical examples [2–7]. An impulsive signal often contains important equipment status information and is also important for system maintenance and process automation. Thus, detecting impulsive signals is of great engineering practical significance, and this has attracted the attention of many researchers.

However, impulsive signals are frequently overwhelmed by strong noise when the fault is at its early engine phase. The impulsive signals may be filtered out because the impulsive energy of the incipient anomaly is very small and the transmission channel is frequently complex. Numerous attempts have been made to extract useful information from such response signals. For example, envelope analysis is widely used [8–11]. Lou and Loparo [8] employed wavelet envelope analysis in the fault diagnosis of rolling bearings and claimed that the decomposed details are different in magnitude between the inner race fault and normal condition. Yu *et al.* [9] applied the EMD and Hilbert method to extract the envelope signal of rolling bearings and found that the fault characteristics can be extracted by selecting proper IMFs. A morphological filter is also an efficient tool in processing impulsive signals. Jing [1] proposed an improved morphological filter for the feature extraction of impulsive signals in the time domain, which aims to extract the entire signal including fault frequency, natural frequency and decay coefficient. Laplace wavelet correlation filtering (LWCF), which uses a Laplace wavelet as the transient model and identifies the parameters by correlation filtering, is effective in detecting a single transient [10]. However, these methods are not suitable for extracting signal features of impulsive signals with strong background noise.

With the aid of stochastic resonance, the unavoidable noise can, however, be applied to enhance the signal-to-noise ratio (SNR) of a system's output. Although the SR method uses random noise to enhance the useful signal characteristics, the signals after SR is often distorted into rectangular-like waves by its nonlinear amplification, which confines the use of SR in the quantitative diagnosis of machinery. Li [11] presented an inversion method to restore the output waveform after SR. However, it is still necessary to locate the inflection point of the system and carry out corresponding special treatment. The program is complicated and requires adjustment of various parameters.

To overcome the reviewed problems, the present study concentrates on developing a more effective technique for extracting impulsive signals with strong noise contamination that cannot be extracted using a morphology filter or other filters. The fault period and phase of impulsive signals are extracted after SR. However, there are still two problems with the SR recovery process. The first problem is intercepting data that contain only a single period of impulsive signals. The recovery process for impulsive signals is different from that of cosine signals or others because of the compact characteristic of impulsive signals in the time domain. To realize the piecewise fitting of data, a moving least squares fitting operation is applied to segment data in the time domain using a sliding window. Using the fault period and phase obtained in the first SR, we can intercept the data that contain only single shocks of attenuation signals. A second stochastic resonance with the intercepted data is then used to obtain damping of the oscillation frequency of the signal to improve inversion results. The experimental results show that the background noise is suppressed effectively and the key features of impulsive signals are reconstructed with good degree of accuracy, which leading to an accurate diagnosis of faults on bearings undertaking a run-to failure test.

## 2. Stochastic Resonance and Impulsive Signal Recovery

### 2.1. The Theory of Stochastic Resonance

Stochastic resonance (SR) is a nonlinear physical phenomenon where weak signals are enhanced and the noise is weakened through the interaction of a small parameter signal and noise for a nonlinear system model. SR is widely used in the extraction of weak cosine-like signals. However, impulsive signals are more common in mechanical systems. In this section the Kramers rate is used to explain the SR characteristic of impulsive signals.

The over-damped motion of a Brownian particle in a bistable potential in the presence of noise and periodic force is considered to describe SR, as in Equation (1) [12,13]:

(1) $$\frac{\text{d}x}{\text{d}t}=-\frac{\partial U}{\partial x}+s(t)+n(t)$$

where *s*(*t*) is the input weak periodic signal which should be detected with the frequency of *f*_0_. Let 〈 *n*(*t*),*n*(*t*+*τ*)〉 = 2*Dδ*(*t*), where *D* is the noise intensity and *δ*(*t*) represents a Gaussian white noise with zero mean and unit variance. Then, Equation (1) can be written as Equation (2):

(2) $$\frac{\text{d}x}{\text{d}t}=ax-bx^{3}+A_{0}s(f_{0}t+\varphi )+\sqrt{2D}\delta (t)$$

where *a* and *b* are barrier parameters of bistable model and are positive real parameters, *A*_0_ is the periodic signal amplitude and *f*_0_ is the driving frequency. The potential function is denoted by $U(x)=-\frac{1}{2}ax^{2}+\frac{1}{4}bx^{4}$.

The crucial process of using SR to detect weak signals is to adjust the interaction of signal and noise to let the Brownian particle jump freely into the left or right potential. The height of the potential barrier is Δ*U* = *a*^2^/(4*b*). In this paper, a normalized scale transformation is applied to enable the classical SR approach to detect signals with large parameters like in [13].

### 2.2. Nonlinear Distortion Phenomenon of SR

**Figure 1.**
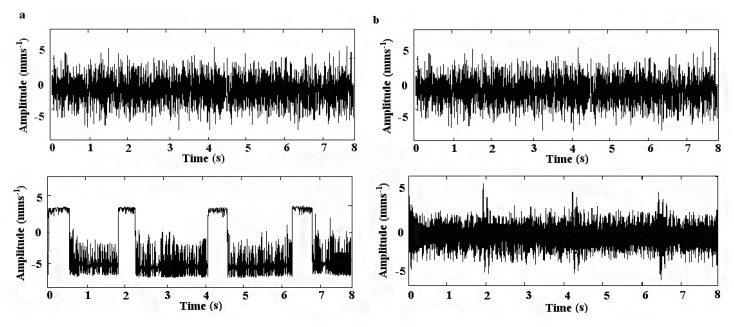
Resonance characteristics of impulsive signals: (**a**) the trapezoidal wave of SR output and (**b**) impulse output of SR.

Although the SR method uses random noise to enhance the useful signal characteristics, the signals after SR is often distorted into rectangular-like waves by its nonlinear amplification, which confines the use of SR in the quantitative diagnosis of machinery. To realize the quantitative diagnosis of rotating machine faults, we need an accurate value for the amplitude of impulsive signal. Hence, we need to study the inversion method for SR. The trajectory of the Brownian particle excited by an impulsive signal can show two different forms of output waves when SR occurs: one is a trapezoidal wave and the other is the form of impulses as shown in Figure 1a,b. As the trapezoidal waveform cannot be easily controlled, and its corresponding SR evaluation index is difficult to select, this resonance form is frequently neglected in practice. As shown in Figure 1a,b, a trapezoidal wave can be used to clearly judge the SR results, and it is more exact when evaluating the information pertaining to fault frequency and phase. In this paper, an adaptive algorithm is applied to enable the classical SR approach to detect the signals concerned in [14,15].

### 2.3. The Recovery of Impulsive Signals

Stochastic resonance can qualitatively judge whether there is any fault, yet it is not suited for quantitative diagnosis because of the nonlinear amplification due to SR. To realize the quantitative diagnosis of rotating machine faults, we need an accurate value for the amplitude of impulsive signal. Hence, we need to study the inversion method for SR.

Traditional curve fitting is a global fitting, which is not suitable for impulsive signals. To realize the piecewise fitting of data, a moving least squares fitting operation is applied to data segment in the time domain using a sliding window.

Let *u*(*x*) be an unknown function whose values are known in the Ω calculation domain *N* nodes (*I* = 1, 2… *N*), namely, *U_I_ = u*(*x*_1_). Additionally, in the calculation domain of Ω*_x_*, *u*^h^ (*x*) is used as the approximation of *u*(*x*) and can be represented as the polynomial:

(3) $$u(x)\approx u^{h}(x)=\sum_{i=1}^{m}p_{i}(x)a_{i}(x)=p^{T}(x)a(x)$$

where, *p*(*x*) is the polynomial base vector; *A*(*x*) is the coefficient vector; *m* is the number of fundamentals.

The moving least squares is to get the weighted sum of squares a minimum structure approximation function through the difference between the order and the corresponding node function value, that is to let Equation (4) take the minimum:

(4) $$\;J=\sum_{I=1}^{n}\omega (x-x_{I}){\left[u^{h}(x)-u(x_{I})\right]}^{2}=\sum_{I=1}^{n}\omega (x-x_{I}){\left[\sum_{i=1}^{m}p_{i}(x)a_{i}(x)-u(x_{I})\right]}^{2}$$

where, *n* is the calculation of the node number point; *x* is contained in the domain Ω*_x_* and node related weight function *ω*:

(5) $$P=\left(\begin{array}{cccc} p_{1}(x_{1}) & p_{2}(x_{1}) & \ldots . & p_{m}(x_{1})\\ p_{1}(x_{2}) & p_{2}(x_{2}) & \ldots . & p_{m}(x_{2})\\ \ldots . & \ldots . & \ldots . & \ldots .\\ p_{1}(x_{n}) & p_{2}(x_{n}) & \ldots . & p_{m}(x_{n}) \end{array}\right)$$

(6) $$W(x)=\text{diag}(w_{1}(x),w_{2}(x),\cdot \cdot \cdot ,w_{n}(x)),w_{i}(x)=w(x-x_{i})$$

(7) $$A(x)=\sum_{I=1}^{n}\omega (x-x_{I})p(x_{1})p^{T}(x_{1})=P^{T}W(x)P$$

(8) $$B(x)=P^{T}W(x)$$

(9) $$u={\left[u(x_{1}),u(x_{2}),\cdot \cdot \cdot ,u(x_{n})\right]}^{T}$$

Equation (4) takes the minimum solution of the coefficient vector *a*(*x*):

(10) $$a(x)=A^{-1}(x)B(x)u$$

Substituting Equation (3) with Equation (5):

(11) $$u^{h}(x)=\varphi (x)u$$

Usually a monomial is selected as the base function, such that a one-dimensional space monomial and quadratic basis function, respectively, are as follows:

(12) $$\{\begin{array}{ll} P^{Τ}(x)=[1,x] & m=2\\ P^{Τ}(x)=[1,x,x^{2}] & m=3 \end{array}$$

A two-dimensional space monomial and quadratic basis function are, respectively, as follows:

(13) $$\{\begin{array}{ll} P^{Τ}(x)=[1,x,y] & m=3\\ P^{Τ}(x)=[1,x,y,x^{2},xy,y^{2}] & m=6 \end{array}$$

This paper focuses on impulsive signals, so we choose the following function as the basis function. As to the fit of weak impulsive signals, *ω_s_* is important because, without it, the least squares fitting will not lead to correct results:

(14) $$s(t)=Ae^{-nt}\sin(2\pi \omega_{s}t+\alpha )$$

**Figure 2.**
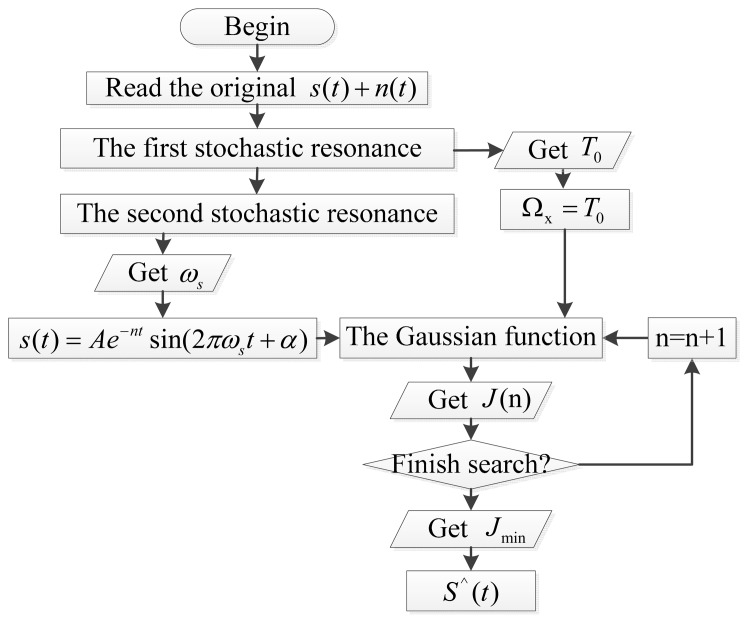
Flow diagram of the recovery of impulsive signals.

In addition, the weight function should: be compact, that is only in the surrounding area is it not equal to zero but in all other areas it is zero; be negative; attenuate, along with the increasing domain |*x* − *x*_I_|, *ω*(*x* − *x*_I_), gradually decay. In this paper, the Gaussian Function (15) is selected as the weight function,

(15) $$\omega_{i}(x)=\left\{\begin{array}{cc} \frac{e^{-\gamma^{2}\beta^{2}}-e^{-\beta^{2}}}{1-e^{-\beta^{2}}} & 0\leq r\leq 1\\ 0 & r>1 \end{array}\right\}$$

where *r* = *d*/*R*_I_, such that *d* is the distance *d* = |*x* − *x*_I_| for calculation point *x*_1_; *R*_I_ is the node domain of the influence radius, *R*_I_ = *k* × *d*_I_; *k* is the radius of the influence multiplier and is slightly greater than 1 to ensure that the calculated point solving domain has enough nodes; *d*_I_ is a dynamic variable that changes with node distribution in dense situations and is smaller when the nodes are concentrated; and *β* is a weighting factor critical for those nodes that are closer to *x* but that has no effect on far nodes. The flow diagram of the recovery of impulsive signals is shown in Figure 2.

### 2.4. Algorithm Construction

The impulsive signal is constructed as:

(16) $$s(t)=x_{0}(t)+n_{0}(t)=\sum_{k}A\cdot \exp[-D_{c}(t-kT_{0}-\tau_{0})]\cdot \sin[\omega_{s}\cdot (t-kT_{0}-\tau_{0})]+n(t)$$

where *A* is the amplitude, *D_c_* is the damping ratio, *k* is the number of impulsive signals, *T*_0_ is the period between different impulsive signals, *τ*_0_ is the time index, *ω_s_* is oscillation frequency and *n*(*t*) is the noise. Considering the compact characteristic of the impulsive signal in the time domain and the need for a moving least squares fitting, we modify two steps of stochastic resonance: the process for extraction and recovery of impulsive signal, which can be described in detail as the following paragraphs.

**Figure 3.**
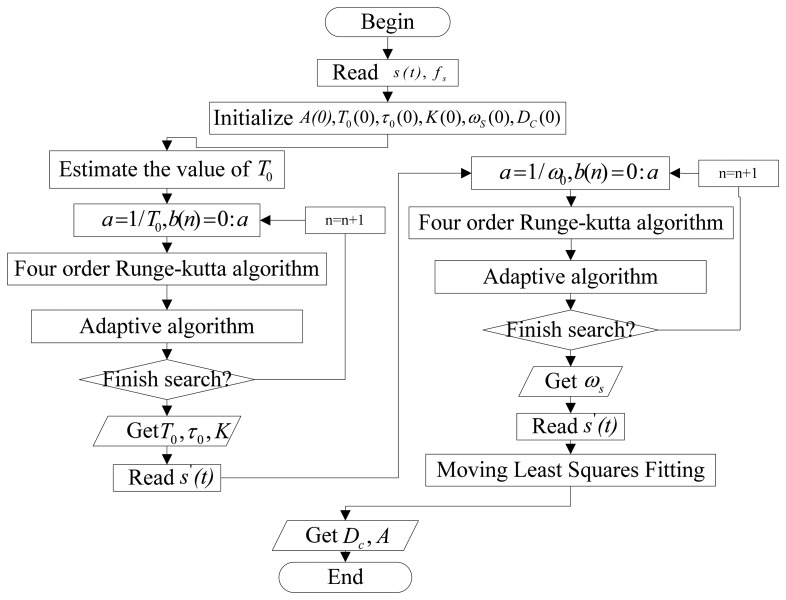
Flow diagram of extraction and recovery of impulsive signals.

First, reading the original signal and the sample rate, the initialization of *A*(0), *T*_0_(0), *τ*_0_(0), *K*(0), *ω_s_*(0), *D_c_*(0) are estimated. These parameters indicate the probable geometric characteristics of the impulsive signal. Then, two steps of adaptive parameter-tuning stochastic resonance are used to update *T*_0_, *τ*_0_, *k* and *ω_s_*, which are shown in Figure 3:

Step 1. Estimate the value of T_0_, let *a* = 1/*T*_0_(0), *b*(*n*) = 0:*a*.
Step 2. According to the adaptive optimization algorithm, we use parameter-tuning stochastic resonance to obtain *T*_0_, *τ*_0_, and *k*.
Step 3. According to the value of *T*_0_ and *τ*_0_, we extract *s*′(*t*) for *K* = 1.
Step 4. Estimate the value of *ω_s_*, let *a* = 1/*ω_s_*(0), *b*(*n*) = 0:*a*.
Step 5. According to the adaptive optimization algorithm, another parameter-tuning stochastic resonance is used to obtain *ω_s_*, which is important because, without it, the moving least squares fitting will not lead to a reasonable result.

Next, the least squares fitting using a Gaussian function as the weight function, the impulsive signal as the interval function and *ω_s_* as the known parameter is used to obtain *A* and *D_c_*. *A* can then be used to realize the quantitative fault diagnosis of the rotating machine.

## 3. Numerical Evaluation

**Figure 4.**
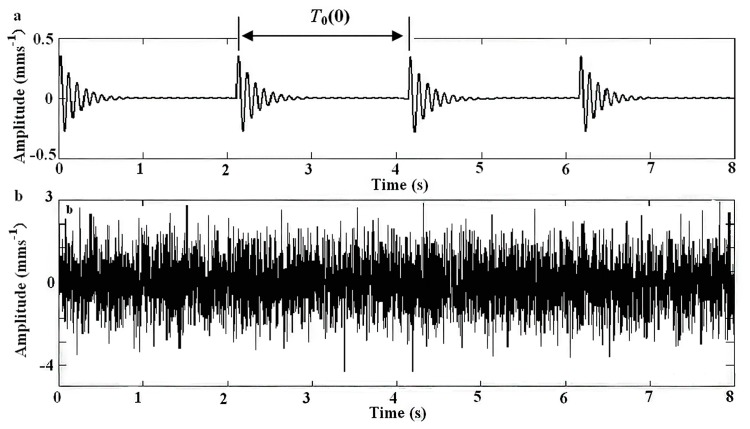
The simulation signal, (**a**) the signal and (**b**) the noisy signal.

To illustrate the effectiveness of the proposed method in detecting and recovering of weak impulsive signals, a simulation is carried out based the signal in Equation (17):

(17) $$s(t)=x_{0}(t)+n_{0}(t)=\sum_{k}A\cdot \exp[-D_{c}(t-kT_{0}-\tau_{0})]\cdot \sin[\omega_{s}\cdot (t-kT_{0}-\tau_{0})]+n(t)$$

where *A* = 0.4 is the signal amplitude, *D_c_* = 5 is the damping ratio, *k* = 4 is the number of impulsive signals, *T*_0_ = 2.1 is the period between different impulsive signals, *τ*_0_ = 0 is the time index, *ω_s_* = 20*π* is the oscillation frequency, *n*(*t*) is the Gaussian noise with a mean value σ = 0 and root-mean-square-value σ = 3, and the sampling frequency is 400 Hz in the time range [0, 8]. Figure 4a presents the simulation signals *x*_0_(*t*) and *n*_0_(*t*) and Figure 4b presents the noisy signal *s*(*t*).

**Figure 5.**
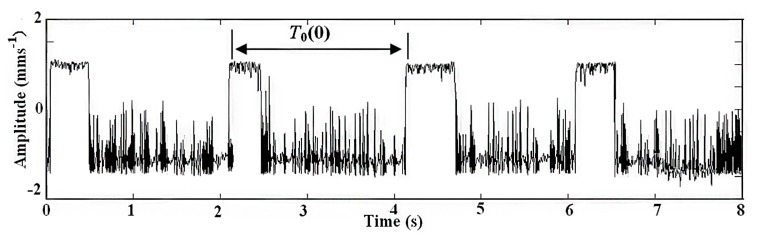
Output of the first parameter-tuning stochastic resonance.

The adaptive parameter-tuning stochastic resonance and moving least-squares fitting is adapted to extract and recover the impulsive signal. Following the previous section's steps, the results obtained by the proposed method from the simulation signal are shown. The result of the first parameter-tuning stochastic resonance is shown in Figure 5, which shows that *T*_0_ = 2.1 s, *k* = 4, *τ*_0_ = 0.

**Figure 6.**
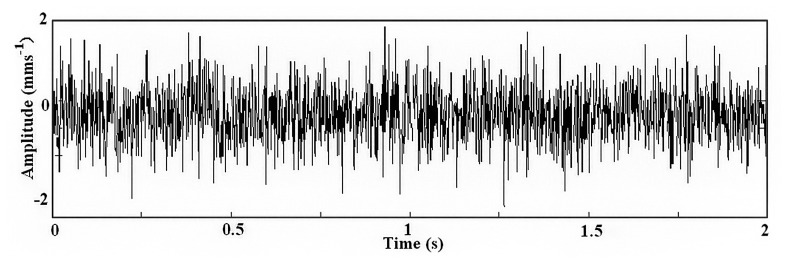
The segment of simulation data.

Then, considering that *T*_0_ = 2.1 s, *k* = 4, *τ*_0_ = 0, a segment of data was segregated in the time range [0, 2] from the above simulation signal as shown in Figure 6.

**Figure 7.**
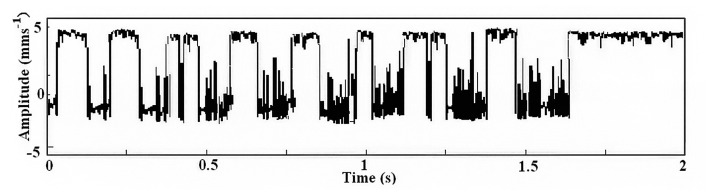
Output of the second parameter-tuning stochastic resonance.

The result of the second parameter-tuning stochastic resonance is shown in Figure 7, and a FFT algorithm is used to find *ω_s_* ≈ 62.83 Hz.

**Figure 8.**
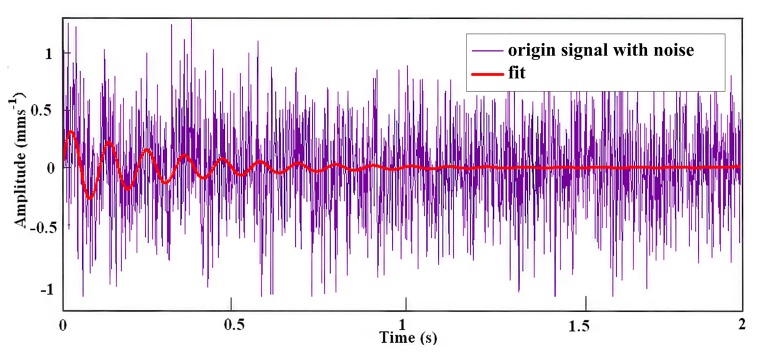
Result of the moving least squares fitting.

The result of the least squares fitting is shown in Figure 8, from which it has found that *A* = 0.38 *mms*^−1^ and *D_c_* = 5.

**Figure 9.**
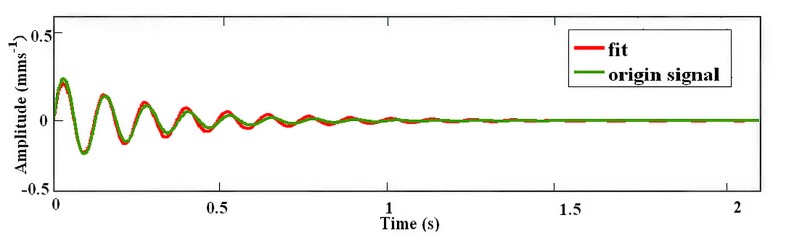
Comparison between the original signal and the least squares fitting.

A direct comparison between the original transient and reconstructed signal is shown in Figure 9. It can be seen they display good agreement.

**Figure 10.**
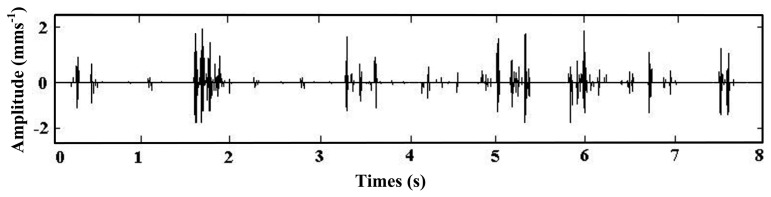
The result of the morphological filtering.

A comparison with morphological filtering is illustrated in Figure 10. A Laplace wavelet is chosen as the structural element of the morphological filtering. It can be found that the impulsive cluster is not extracted from the original because of the influence of strong noise. Additionally, the traditional recovery method for SR does not involve a sliding window, which is vital to the compact characteristic of impulsive signals in the time domain. Thus, the morphological filtering method cannot be used in the recovery of the impulsive signal after SR.

The simulation results demonstrate the effectiveness and superiority of the proposed method in the detection of weak impulsive signals.

## 4. Application in Rotating Machine Fault Diagnosis

**Figure 11.**
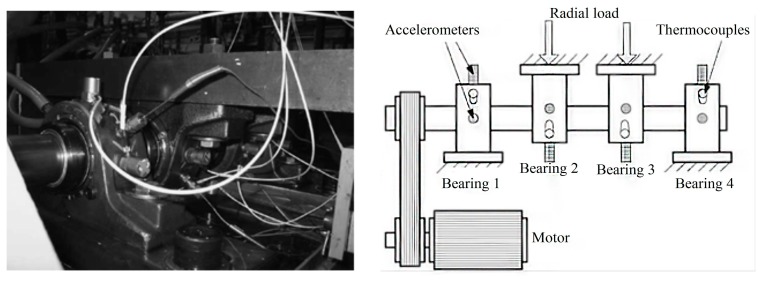
Bearing test rig and sensor placement illustration.

To validate the proposed method a bearing run-to-failure test was performed under normal load conditions on a specially designed test rig. The bearing test rig consisted of four test bearings on one shaft. The shaft was driven by an AC motor and coupled by rub belts. The rotation speed was kept constant at 2000 rpm. A radial load of 2000 N was added to the shaft and bearing by a spring mechanism. All the bearings were lubricated through an oil circulation system which regulates oil flow to maintain constant temperature of the lubricant. Figure 11 shows the test rig and illustrates sensor placement. The failure of all bearings occurred after exceeding the designed lifetime of the bearing, which is more than 100 million revolutions.

A magnetic plug installed in the oil feedback pipe collected debris from the oil as evidence of bearing degradation. The test stopped when the accumulated debris adhered to the magnetic plug exceeded a certain level and caused an electrical switch to close. Four Rexnord ZA-2115 double row bearings were installed on one shaft as shown in Figure 11. The bearings had 16 rollers in each row, a pitch diameter of 7.15 cm, roller diameter of 0.841 cm, and a tapered contact angle of 15.171°. A PCB 353B33 High Sensitivity Quartz ICPs accelerometer was installed on the housing of each bearing. Four thermocouples were attached to the outer race of each bearing to record bearing temperature for monitoring the lubrication. Vibration data were collected every 10 min by a National Instruments DAQ Card-6062E data acquisition card. The data sampling rate was 20 kHz and the data length was 20,480 points. Data collection was controlled by a National Instruments LabVIEW program. The parameters of the experimental bearing are shown in Table 1. BPFI, BPFO, BSF and FTF represent the characteristic frequency of the inner race fault, outer race fault, ball fault and the cage fault, respectively.

**Figure 12.**
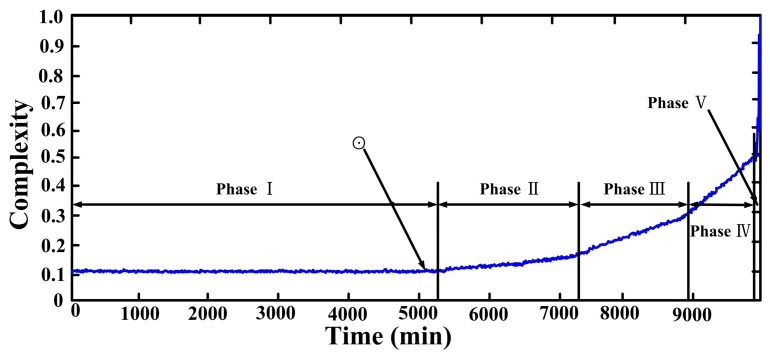
The Lempel-Ziv complexity features for the whole life cycle of the bearing with an outer fault.

Figure 12 shows the monitoring result for the whole test using, which are obtained by using the morphological filter and the time complexity to the vibration data. In [16] the monotone increasing trend of the result has been viewed into five phases to represent different degrees of severity and fault progression. However, it is clear that the morphological filter cannot extract fault information before and near the symbols ⊙ in Phase I.

**Figure 13.**
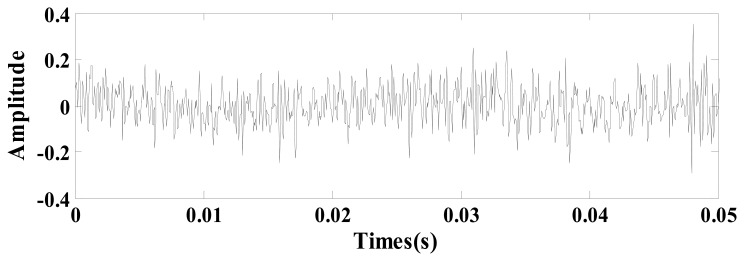
The signal of early outer race fault.

To demonstrate the effectiveness of current method, signals in Phase I are used for applying the adaptive parameter-tuning stochastic resonance and moving least squares fitting. Figure 13 shows a segment of the signal measured at time instant of 5000 min. From it there is no impulsive information which can be observed.

**Figure 14.**
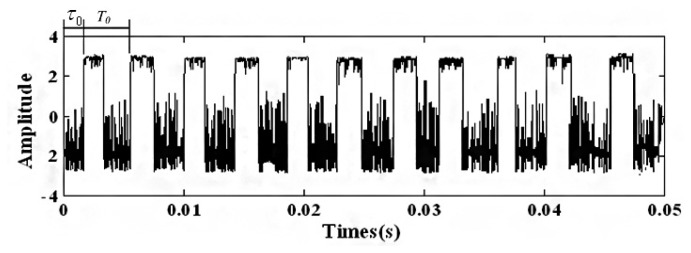
Output of the first parameter-tuning stochastic resonance.

The result of the first parameter-tuning stochastic resonance is shown in Figure 14, from which it has found that *T*_0_ = 0.0038 s, *k* = 4, *τ*_0_ = 0. Equation (18) indicates that the fault frequency has a good match with the characteristic frequency of the outer race fault as Table 1, which preliminarily verifies the effectiveness of the method:

(18) $$f=\frac{1}{T_{0}}=\frac{1}{0.0038}=\frac{1}{0.0038}\approx 263.2\;\text{Hz}$$

**Figure 15.**
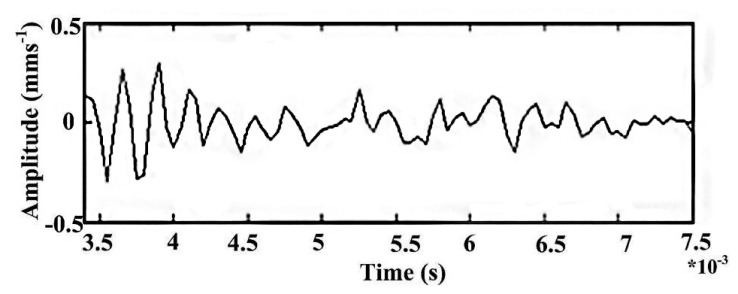
The intercept data of early outer race fault.

Then, considering that *T*_0_ = 0.0038 s, *k* = 4, *τ*_0_ = 0, data in the time range [0, 0.0008] is segregated from the simulation signal as shown in Figure 15.

**Figure 16.**
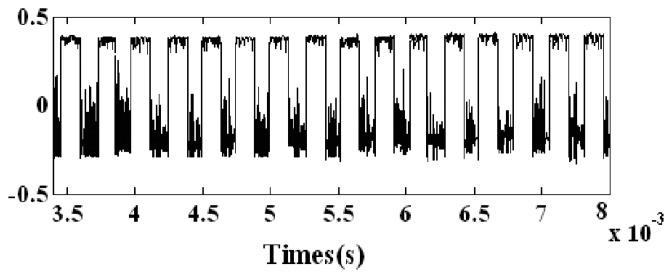
Output of the second parameter-tuning stochastic resonance.

The result of the second parameter-tuning stochastic resonance is shown in Figure 16, *ω_s_* ≈ 3989.8 Hz.

**Figure 17.**
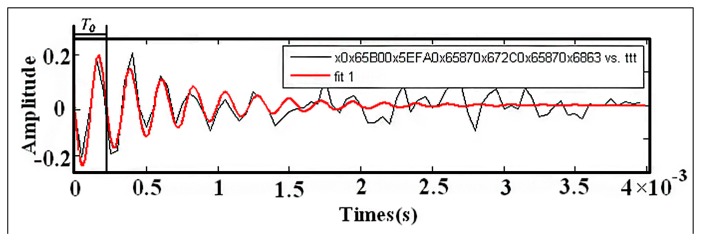
The result of the moving least squares fitting.

The result of the least squares fitting is shown in Figure 17, from which we find *A* = 0.21 and *D_c_* = 5.

**Figure 18.**
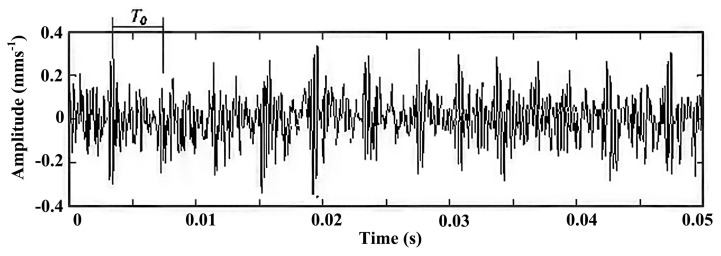
The signal of serious outer race fault.

**Figure 19.**
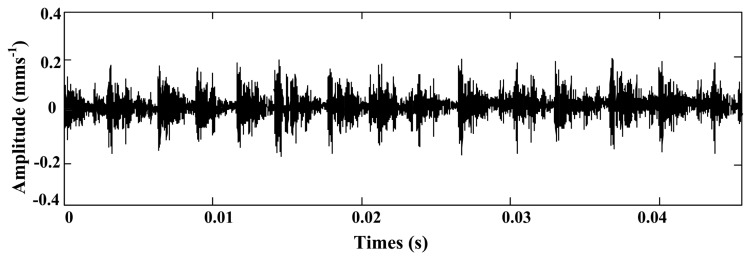
The morphology filtering result of serious outer race fault.

**Figure 20.**
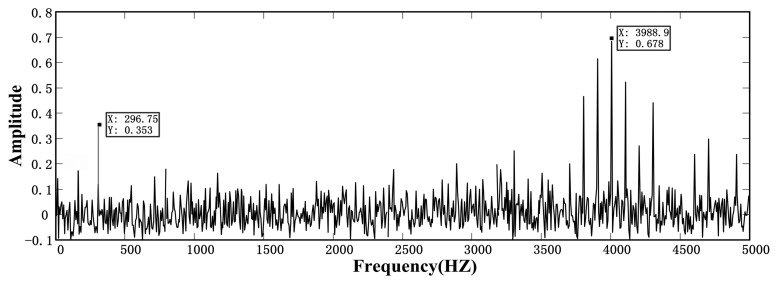
The morphology filtering result of serious outer race fault.

Figure 18 is a section of data intercepted from Phase IV. Figure 19 is the morphology filtering of this data, and Figure 20 is the FFT processing of this data.

From Figure 20, we find that the proposed method has a good match with the frequency of the outer race fault *f* ≈ 263.2 Hz and the oscillation damping vibration frequency *ω_s_* ≈ 3989.8 Hz. The proposed method can predict bearing fault information much earlier than morphology filtering. Moreover, the amplitude of the impulsive signal can be further used to judge the severity of the fault of the rotating machine. Therefore, the method proposed in this study has good processing ability for weak impulsive signals and a specific practical application.

## 5. Conclusions

In this paper, a stochastic resonance (SR)-based method of recovering weak impulsive signals is developed for quantitative diagnosis of faults in rotating machinery. It has been shown in theory that weak impulsive signals fulfill the mechanism of SR, but the SR produces nonlinear distortion of the shape of the impulsive signal. To eliminate the distortion a moving least squares fitting method is induced to reconstruct the signal from the output of SR process. To verify the effectiveness of the proposed method, a contrastive analysis between the proposed method and the morphology filter is conducted based on simulation. Experiments are carried out on bearings with outer race faults to verify the proposed approach. The experimental results show that the background noise is effectively suppressed and the key features of impulsive signals are reconstructed with good degree of accuracy, which leads to an earlier diagnosis of faults in bearings undertaking a run-to failure test.

## Figures and Tables

**Table 1.** Parameters of the experiment bearing.

**Bearing Designation**	**Ball Numbers**	**Groove Section Size (cm)**	**Contact Angle**	**BPFI (Hz)**	**BPFO (Hz)**	**BSF (Hz)**	**FTF (Hz)**
ZA-2155 of Rexnord	16	0.841	2.815	296.9	263.4	139.9	29.55
